# Taxonomically restricted genes are associated with the evolution of sociality in the honey bee

**DOI:** 10.1186/1471-2164-12-164

**Published:** 2011-03-29

**Authors:** Brian R Johnson, Neil D Tsutsui

**Affiliations:** 1 Department of Environmental Science, Policy & Management University of California, Berkeley 137 Mulford Hall, MC3114 Berkeley, CA 94720-3114 USA

## Abstract

**Background:**

Studies have shown that taxonomically restricted genes are significant in number and important for the evolution of lineage specific traits. Social insects have gained many novel morphological and behavioral traits relative to their solitary ancestors. The task repertoire of an advanced social insect, for example, can be 40-50 tasks, about twice that of a solitary wasp or bee. The genetic basis of this expansion in behavioral repertoire is still poorly understood, and a role for taxonomically restricted genes has not been explored at the whole genome level.

**Results:**

Here we present comparative genomics results suggesting that taxonomically restricted genes may have played an important role in generating the expansion of behavioral repertoire associated with the evolution of eusociality. First, we show that the current honey bee official gene set contains about 700 taxonomically restricted genes. These are split between orphans, genes found only in the Hymenoptera, and genes found only in insects. Few of the orphans or genes restricted to the Hymenoptera have been the focus of experimental work, but several of those that have are associated with novel eusocial traits or traits thought to have changed radically as a consequence of eusociality. Second, we predicted that if taxonomically restricted genes are important for generating novel eusocial traits, then they should be expressed with greater frequency in workers relative to the queen, as the workers exhibit most of the novel behavior of the honey bee relative to their solitary ancestors. We found support for this prediction. Twice as many taxonomically restricted genes were found amongst the genes with higher expression in workers compared to those with higher expression in queens. Finally, we compiled an extensive list of candidate taxonomically restricted genes involved in eusocial evolution by analyzing several caste specific gene expression data sets.

**Conclusions:**

This work identifies a large number of candidate taxonomically restricted genes that may have played a role in eusocial evolution. This work thus lays the foundation for future functional genomics work on the evolution of novelty in the context of social behavior. We also present preliminary evidence, based on biased patterns of gene expression, that taxonomically restricted genes may have played a role in the evolution of caste systems, a characteristic lineage specific social trait.

## Background

Division of labor, a hallmark of advanced insect sociality, has long attracted attention from biologists, as these systems of polyphenism are some of the most elaborate in the animal kingdom [1-3]. The honey bee, for example, has queen and worker physical morphs, along with 4 physiologically-based worker castes [4-6]. Great progress has been made in recent years towards unraveling how changes in the regulation of juvenile hormone and vitellogenin underlie these adaptively regulated differences in phenotype [3,6]. These successes have strengthened the view that changes to an insect groundplan (conserved sets of genes organized into stable networks that underlie traits such as reproductive behavior) are central to eusocial evolution [7-11].

Although changes to an insect groundplan are undoubtedly a major force in eusocial evolution, it is likely that other mechanisms have also played a role in the evolution of distinctive eusocial traits. In particular, we hypothesize that the origin of eusociality may have involved novel genes. If so, many of these new genes would likely be taxonomically restricted genes (TRGs), orphan sequences not found outside a species or only found in a particular taxonomic group [12-17]. TRGs make up ~10-20% of the genes in every genome sequenced to date [16]. In *Drosophila melanogaster*, the species with the most complete and accurate genome, TRGs make up approximately 18.6% of genes [18]. In addition to being abundant, TRGs are important for generating lineage specific traits [16,19]. In *Drosophila*, for example, novel courtship behavior was found to be partially dependent on a novel gene, sphinx [19]. Likewise in *Hydra*, changes in the expression of a TRG are important for species specific differences in tentacle formation [20]. Finally, species that rely on toxins for predatory behavior or defense possess specialized venoms with taxonomically restricted occurrence [21].

Here we explore for the first time, using comparative genomics, whether TRGs could have played a broad role in eusocial evolution. Honey bee biology allows for a simple test of this possibility. The honey bee queen is characterized by a sharp reduction in behavioral and morphological complexity [22]. The behavior of the queen is largely limited to fighting other queens and laying eggs, while her morphology is missing many traits present in the workers [22]. The workers, in contrast, exhibit highly derived lineage-specific behavior thought to have evolved since the origin of eusociality. Workers, for example, feed the larvae a specialized secretion, build the complex nest, and forage cooperatively in a system dependent on advanced communication not seen in their solitary ancestors [23,24]. If TRGs are important for generating the expansion of behavioral complexity associated with the evolution of eusociality, we predict that TRGs limited to eusocial species should be disproportionately associated with the worker caste relative to the queen case.

This study has four goals. First, we test the hypothesis that TRGs are associated with eusocial evolution by analyzing an extensive list of genes that are differentially expressed between honey bee castes [25,26]. Second, we explore the frequency and characteristics of TRGs in the honey bee genome. We classify each gene in the official gene set into a TRG category and then determine basic characteristics of these genes, such as protein size, exon number, transcript support, and so forth. We then use Interproscan [27] to functionally annotate as many TRGs as possible. We also reevaluate the nature of insect-specific proteins, incorporating data from several recently sequenced insect and arthropod genomes. Third, we test whether TRGs (orphans or those limited to the hymenoptera) are expressed in a gland known to be involved in a derived eusocial behavior. Finally, we generate a list of candidate TRGs potentially involved in eusocial evolution. These are TRGs associated with the evolution of either queen/worker dimorphism, a gland specialized for a novel function, or division of labor amongst the workers.

## Results and Discussion

### TRGs in the honey bee official gene set 2

The honey bee official gene set is biased towards conserved genes, as conservation was a factor in determining which gene predictions to include [28]. Our purpose in this section is therefore not to quantify the absolute abundance of TRGs in the honey bee genome, as this analysis awaits characterization of the many gene predictions that are not in the official gene set. Our goals, in contrast, are to determine (1) if there are large numbers of TRGs within the official gene set, and (2), to identify the characteristics and functions of these TRGs. In short, we used blastp and tblastn along with the protein sets and genomes of 27 species (23 insects, tick, human, *C. elegans*, and *S. cerevisiae*) to search for homologs for each honey bee gene in each of the species, using an E value of 10^-4 ^as the cutoff [12-15]. The results of this analysis were used to construct a database to which queries were made to identify TRGs. Genes identified as TRGs were then blasted against the current NCBI nr protein database to ensure their correct homology classification. TRGs were then interrogated with Interproscan to functionally annotate as many as possible.

**Figure 1:**
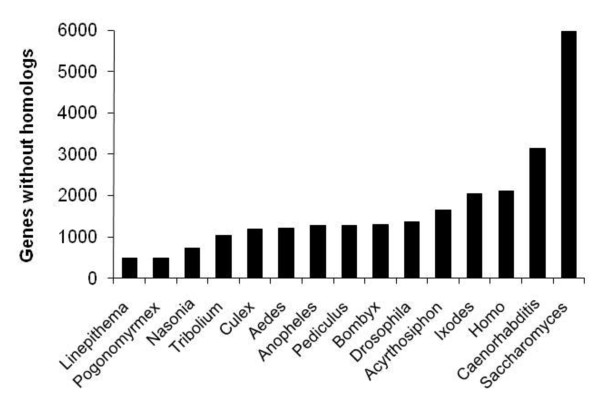
**Number of genes in honey bee OGS2 without homologs in other genomes**. Although all 12 *Drosophila *genomes were used, only *Drosophila melanogaster *is shown because all *Drosophila *species had very similar numbers of homologs with *Apis mellifera*. Number of genes without homologs roughly followed taxonomic relatedness with the closely related ants having homologs for the largest number of *Apis mellifera *genes.

**Figure 2:**
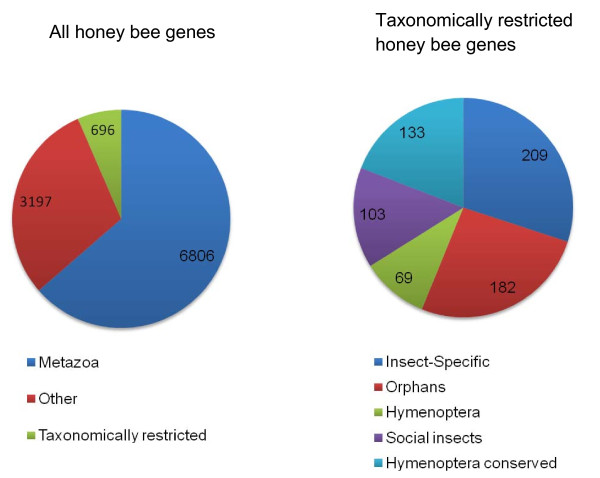
**Percentages of *Apis mellifera *genes in taxonomically and non-taxonomically restricted categories**.

Figure 1 shows the number of genes in the honey bee official gene set 2 (OGS2) without homologs in each of the other species. The number of such genes varies roughly with taxonomic distance, with closely related species tending to have more homologs and distantly related species fewer. Figure 2 shows the breakdown of TRGs in OGS2. 696 genes were TRGs, 6806 were present in all metazoans, and 3197 showed other patterns of occurrence. Among the TRGs, we identified 5 classes (see methods for definitions): orphans (182 genes), Hymenoptera (69 genes), social insect (103 genes), Hymenoptera-conserved (133 genes), and insect-specific (209 genes). Table 1 shows the characteristics of genes in each taxonomic category. As for orphans in other species such as *Drosophila *and primates, honey bee orphans are shorter than highly conserved sequences, have fewer exons, and fewer of them have transcript support. Thus, as per previous studies, it is possible that honey bee TRGs (orphans and genes limited to the Hymenoptera) are young genes formed via a variety of mechanisms such as gene duplication, exaptation from transposable elements, horizontal transfer, and *de novo *gene formation [14,29]. Future work on the identified TRGs will explore these questions.

**Table 1:** Gene characteristics of taxonomically restricted genes

	Protein Size (aa)	Exon Number	Gene Size (nt)	GC Content (cds)	Transcripts
	mean ± SE	mean ± SE	mean ± SE	mean ± SE	%
Orphans	142.55 ± 14.62	2.41 ± 0.10	2191.47 ± 440.52	36.89 ± 0.70	45.05
Hymenoptera	190.07 ± 19.97	2.89 ± 0.16	2690.57 ± 440.54	39.21 ± 1.29	51.32
Social Insect	314.62 ± 28.18	3.35 ± 0.18	7638.89 ± 4634.22	39.02 ± 1.03	47.57
H-conserved	366.61 ± 34.65	3.20 ± 0.16	3625.76 ± 314.46	41.50 ± 1.03	54.14
Insect	431.40 ± 25.27	5.35 ± 0.36	4456.40 ± 577.34	41.81 ± 0.60	81.43
Metazoa	620.15 ± 7.91	7.47 ± 0.08	10266.52 ± 354.80	38.30 ± 0.11	91.92

H-conserved stands for Hymenoptera-conserved, while Insect stands for Insect-Specific.

The mean size of orphans in the present study is larger than that found in many previous studies of orphans [12-15], reflecting the probability that the official gene set is missing many orphans. There are additional reasons to suspect that the actual number of TRGs (in all taxonomic classes) in the honey bee genome is significantly higher than that found within the official gene set. First, *Drosophila melanogaster*, with a better assembled genome, and most other sequenced insects, have approximately 10-20% TRGs [12,16,18]. Second, inclusion in the honey bee official gene set was partly based on homology with other sequences. Hence, thousands of gene predictions were not included in the official set, which is only 10,699 genes (a small number compared to most other holometabolous insects). Further, when we manually aligned ESTs from the brain cDNA library used by Grozinger et al [25] to gene predictions not in the official gene set, we were able to unambiguously identify 268 genes. As the cDNA libarary is from the brain alone, it is likely that many of the gene predictions not in the official gene set are real genes that will be annotated when more tissue specific gene expression studies are conducted in the honey bee.

As expected, few of the TRGs in the highly restricted categories (orphans, Hymenoptera, Hymenoptera conserved, and social insect) have been the focus of experimental work (18 genes) or could be characterized with Interproscan (3 genes). However, the functions of many of these genes are consistent with the hypothesis that highly restricted TRGs are associated with lineage specific traits, including eusocial behavior. Most of the named TRGs fell into a few classes: venoms, cuticle proteins, silk proteins, immune genes, and odorant binding proteins. Although these genes are likely associated with both lineage specific traits in general, and eusocial evolution, odorant binding (olfactory behavior in the context of pheromone based communication) and immunity (defense again pathogens in the crowded hive) are thought to be associated with unique selection pressures in the context of eusocial evolution [30,31]. That many TRGs found only in the Hymenoptera are involved in novel behavior is encouraging and suggests that the other TRGs in these categories are interesting candidates for genes underlying lineage specific and or novel eusocial traits. Table 2 lists all named or functionally annotated TRGs.

**Table 2:** Taxonomically restricted genes with names or functions based on Interproscan

OGS2 ID	Name	Function	Homology Class
GB16929	none	cell cycle arrest	Social Insects
GB10975	none	methylation	Hymenoptera
GB12636	Apidermin 3	Cuticle protein	Hymenoptera-conserved
GB10737	Apidermin 2	Cuticle protein	Orphan
GB13172	none	Immune system	Orphan
GB18323	Abaecin	Immune system	Hymenoptera
GB17538	Hymenoptaecin	Immune system	Hymenoptera-conserved
GB20134	OBP 2	Odorant binding	Hymenoptera
GB19454	OBP 3 & OBP 7	Odorant binding	Orphan
GB13299	OBP 12	Odorant binding	Social Insects
GB30438	none	Respiratory electron transport chain	Hymenoptera
GB12184	Silk fibroin 3	Silk + unknown	Hymenoptera
GB12348	Silk fibroin 4	Silk + unknown	Hymenoptera
GB17818	Silk fibroin 1	Silk + unknown	Hymenoptera
GB19585	Silk fibroin 2	Silk + unknown	Hymenoptera
GB15233	AmelSA1	Silk glue + unknown	Hymenoptera
GB19468	Apisimin precursor	brood care	Orphan
GB10355	Melittin precursor	Venom	Hymenoptera
GB19804	Secapin	Venom	Hymenoptera
GB13285	Mast degranulating	Venom	Orphan
GB18161	Apamin	Venom	Orphan

### Insect Specific Proteins

We characterized 209 proteins as insect specific, found in all insects but in no other groups. Zhang et al [13], using different methods, identified a smaller number of such genes, 51. There is nearly no overlap in the insect specific genes found in these two studies [Additional file 1: Supplemental Table S1]. There are three reasons for this difference. First, Zhang et al required extensive coverage as well as a significant blast hit for homology. This is an unusual requirement that has not been used in other studies, probably because distant homologs, such as those between insects and mammals, often have low coverage scores even when they have extremely low E values. Thus, most of the genes identified by Zhang et al [13] as insect specific were classified as metazoan specific in this study, as they had strong blast hits across all tested groups. Second, because the previous study required a high coverage score for homology between insect genes, many genes that could be classified as homologs were left off of the list. Hence, the list of insect specific genes in the present study is larger. Finally, no arthropod genomes, other than those of insects, had been sequenced when the previous study was performed and many genes identified as insect specific were found to be arthropod specific, as they are found in arachnids (tick) or crustaceans (*Daphnia*). Cuticle proteins and hemocyanins are two classes of genes that fall into the category of arthropod specific. The Interproscan analysis of the insect specific genes found in the present study, using honey bee genes as the queries, found that nearly half (45.9%) are odorant binding proteins, 34.0% are uncharacterized, with the rest falling into numerous categories [Additional file 2: Supplemental Table S2].

### Role of gene duplications in generating Hymenopteran TRGs

**Figure 3:**
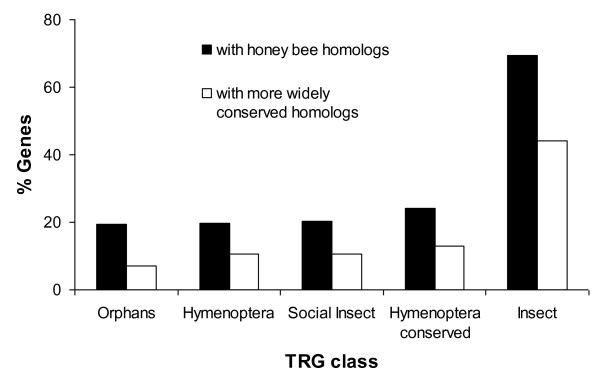
**Percentages of *Apis mellifera *genes in taxonomically restricted categories with evidence for origin via gene duplication**. A small percentage of genes in the most taxonomically restricted categories have paralogs that show homology to genes outside their taxonomic class. Hence, there is little support for most of these genes having formed via gene duplication followed by rapid sequence divergence. A much higher percentage of insect specific genes could, however, have evolved in this fashion, as a high percentage of such genes have paralogs that are widely conserved.

Gene duplication followed by rapid sequence divergence in one of the paralogs is a well explored mechanism for generating novel genes (29, 32). A simple mechanism for identifying such cases is to blast a TRG against all the genes within the same genome and determine whether any of the gene's paralogs are widely conserved (14). We conducted such an analysis with TRGs in all categories to determine a minimum percentage of these genes that could have evolved via gene duplication. Figure 3 shows that relatively few (6.04-12.88%) of the TRGs in the most taxonomically restricted categories (orphans, Hymenoptera, social insect, Hymenoptera-specific) could be shown to have evolved by gene duplication. Insect specific genes were much more likely to be part of a gene family with members that are homologous with genes found outside the insects (43.6% of genes). A list of all TRGs with parent genes in the honey bee genome that are widely conserved is provided [Additional file 3: Supplemental Table S3].

### TRGs in eusocial evolution

Grozinger et al [25] determined gene expression in 5547 ESTs in the brains of queens and workers. After aligning these ESTs to genes, we identified 663 genes with higher expression in queens, 537 with higher expression in workers, and 1880 that were not differentially expressed. We then determined the homology classification of each gene in all three sets. We found that homology status and differential gene expression were not independent (Chi Square Test: *χ*^2 ^= 17.81, *df *= 2, *p *< 0.001; Table 3). Given that the largest percentage deviation came from TRGs expressed in the worker caste (82.7%), we conclude that TRGs were overrepresented amongst genes with higher expression in workers relative to those that were not differentially expressed or had higher expression in queens. Most of the orphans in all three groups (worker, queen, or non-differentially expressed) came from gene predictions not included in the official honey bee gene set. These genes, however, all have transcript support and are almost certainly real genes. We repeated this analysis with different cut-off p-values (for determining significant differences in gene expression) and found that this pattern remains significant down to a p-value of 0.001 (below which there are insufficient data for meaningful comparisons). A table with these additional analyses is presented in the supplemental information [Additional file 4: Supplemental Table S4].

**Table 3:** Taxonomically restricted genes associated with division of labor or glandular specialization

Grozinger et al 2007	Queens	%	Workers	%	No difference	%
Orphan	18	2.71	37	6.88	61	3.24
Hymenoptera	1	0.15	2	0.37	7	0.37
Social-Insect	5	0.75	5	0.93	10	0.53
Hymenoptera-Conserved	5	0.75	4	0.74	17	0.90
Insect	10	1.51	7	1.30	15	0.80
Metazoa	464	69.98	367	68.22	1339	71.22
Other	160	24.13	116	21.56	431	22.93
Total	663		538		1880	
						
Alaux et al (2009)	**Nurses**	%	**Foragers**	%		
Orphan	21	2.39	16	3.17		
Hymenoptera	4	0.46	5	0.99		
Social-Insect	4	0.46	5	0.99		
Hymenoptera-Conserved	6	0.68	4	0.79		
Insect	10	1.14	6	1.19		
Metazoa	564	64.16	326	64.68		
Other	270	30.72	142	28.17		
Total	879		504			
						
	**HP Glands**	%				
Orphan	2	1.89				
Hymenoptera	0	0.00				
Social-Insect	1	0.94				
Hymenoptera-Conserved	1	0.94				
Insect	1	0.94				
Metazoa	57	53.77				
Other	44	41.51				
Total	106					

We also looked for differences in the number of TRGs differentially expressed in the brains of nurses and foragers [27]. Although foragers have more complex behavior than nurses, a prediction is not straight forward because although nurse bee behavior is simple, it is also quite derived [6]. Alaux et al [27] determined gene expression in 1439 ESTs in nurses and foragers. After aligning ESTs to genes from this study, we identified 879 genes with higher expression in nurses and 504 with higher expression in foragers. We found that TRGs did not occur with different frequency between nurses and foragers (Chi Square Test: *χ*^2 ^= 0.49, *df *= 1, *p *= 0.48).

Three major innovations characterize eusocial evolution: division of labor, advanced communication systems, and an expanded task repertoire (7-9, 24). We focused on the third innovation, which is poorly understood at the genetic level. We found that orphans are disproportionately associated with the worker caste relative to the queen caste. This suggests that novel genes may have been important in generating the lineage specific eusocial traits that characterize task repertoire expansion, because workers perform more derived and novel tasks than queens. This initial analysis is based on patterns of gene expression in the brain alone. Future work on more tissues should shed light as to how widespread this pattern is in bees and other social insects. Future work will also assess which of the novel genes are associated with lineage specific traits having little to do with eusocial evolution, and which are associated with novel eusocial tasks such as complex nest building, nestmate recognition, and pheromone production.

### Role of TRGs in a gland specialized for a social function

The hypopharyngeal gland (HPG) is specialized for caste specific behavior in worker honey bees [6,22]. The genes expressed in this gland are therefore of interest for determining what role TRGs may have played in eusocial evolution. The genes from a cDNA library constructed from HPG ESTs supplied by the Robinson Lab, was therefore analyzed to search for candidate TRGs potentially involved in lineage specific brood care, or other caste specific behaviors associated with this gland. Overall, 7 orphans or TRGs found only in the Hymenoptera (out of 106 genes total that could be identified from ESTs) are highly expressed in the hypopharyngeal gland. Only one has a name, *Apisimin*, an orphan known to be an important component of royal jelly [33]. A list of all those ESTs that could be aligned to genes from the hypopharyngeal gland are provided in the supplemental information along with the classification of each gene [Additional file 5: Supplemental Table S5].

The analysis of the genes highly expressed in the hypopharyngeal gland showed that TRGs make up a relatively small portion of expressed genes. However, many of the genes in the full list are clearly housekeeping genes and the important question concerns the genetic basis of the novel eusocial traits associated with this gland. The gland secretes a proteinacious brood food, and caste development signal, in nurse bees, and produces enzymes necessary for conversion of nectar to honey in older bees [6,34]. The nature of the brood food is well understood. Most of the protein content of this secretion is composed of royal jelly proteins, derived members of the yellow protein family [35]. Hence, the genetic basis of most of this gland's specialized secretion is not based on TRGs. However, the number of royal jelly proteins in an insect genome is not conserved and the number in the honey bee could have expanded in response to selection for specialized brood food. The difficultly with this hypothesis is that *Nasonia*, a solitary wasp, also has many royal jelly proteins. However, *Nasonia's *royal jelly proteins appear to be closer in relationship to one another than to those of the honey bees, suggesting they have a different origin [36]. Hence, novel genes (albeit associated with the expansion of a gene family rather than orphans) could have been central to the evolution of this case of specialization. Finally, at least 1 orphan, *Apisimin*, plays an important role in the hypopharyngeal gland, by interacting with the royal jelly proteins [33]. Given that we identified several other TRGs expressed in this gland, it is possible that TRGs have also been important for this case of eusocial evolution.

### Candidate TRGs involved in eusocial evolution

Based on gene expression differences between workers and queens, nurses and foragers, and expression in the HP gland, we identified 146 TRGs (orphans or genes found only in the Hymenoptera) that are candidates for playing a role in eusocial evolution [Additional file 6: Supplemental Table S6]. 7 TRGs with bioinformatically inferred functions thought to be associated with eusocial evolution were found in this data set. One gene, apisimin, experimentally shown to be involved in eusocial evolution in the context of specialized brood care, is in this data set. This suggests that experimental characterization of the other candidates should be productive. Perhaps the most interesting pattern is that three TRGs associated with silk production in larva were found to differ in expression between adults.

Silk proteins in the honey bee, like those in several other groups of insects, are highly diverged, and probably have an independent origin from other insect silks [37]. Three of the five genes underlying silk production were found in the gene set of proteins differing between nurses and foragers (GB12085, GB15233, and GB17818). This is surprising, as silk is used by larva for cocoon construction. There are two possible solutions for this paradox. One, either the silk genes are not expressed in the brain, and are actually expressed in the labial glands, the glands that produce silk in the larva, or these genes have evolved radically different functions in the adult brain. Micro-dissection of insect brains is not error free and it is known that contamination from the hypopharyngeal glands occurs [25,27]. Although speculative, it is possible that material from the labial glands, which are also in the head capsule, are present in brain dissections. If so, then perhaps the silk proteins and silk glue could have evolved a function related to pheromone deposition or transmission, as the labial glands may be involved in pheromonal communication in adult honey bees [38].

### Relationship to previous studies of eusocial evolution

The honey bee has been the subject of a number of studies seeking to understand the genetic basis of particular traits known to be important for eusocial evolution (30-31, 33-35, 37). Viljakainen et al (31) found rapid evolution in immunity genes, for example, while Bilikova et al (33) found a novel protein involved in brood care, *Apisimin*. Likewise, studies have found increases or decreases in gene families known to be involved in eusocial evolution (30, 35). This study identified all of the previously discovered novel genes, along with many more candidates for future study. This work hence builds on previous research by suggesting how important novel genes may be across the genome. In the most relevant previous study, Hunt et al (10) found that genes with higher expression in queens have higher evolutionary rates than genes with higher expression in workers. At first glance, this appears to contradict the results of the present study, which is that genes with higher expression in workers are more likely to be novel, or taxonomically restricted. However, a simple possible explanation for the difference between the two studies is that although queens do not exhibit novel traits, they do exhibit highly derived versions of ancestral traits (related to reproduction). Workers in contrast do novel things altogether. Hence, we could expect derived versions of the same genes in queens and different genes altogether in workers. This hypothesis is speculative, of course, and will need to await verification in future studies.

## Conclusions

Social insects have gained many new morphological and behavioral traits relative to their solitary ancestors [reviewed 24, 39]. The task repertoire of an advanced social insect can be 40-50 tasks, about twice that of a solitary wasp or bee [40]. Our work identifies TRGs that may play a role in the evolution of such lineage specific traits. First, we found support for our hypothesis that highly restricted TRGs should be disproportionately associated with the worker caste relative to the queen caste because workers exhibit the novel behavior of honey bees. Second, our analysis of homology patterns of genes within the official gene set found about 500 orphans or TRGs found only in the Hymenoptera (orphans, Hymenoptera, social insect, Hymenoptera-conserved). Many of those that have been the focus of experimental work are associated with novel eusocial traits. Finally, we compiled an extensive list of candidate TRGs that could be associated with the evolution of eusociality. This work should facilitate and encourage further work on the role played by TRGs in eusocial evolution.

## Methods

### Sequence data sets

A total of 27 genomes were used in this study. The *Apis mellifera *protein set (OGS2) and abinitio gene predictions were downloaded from BeeBase. Ant protein sets and genomes (*Linepethelia *and *Pogonomyrmex*), were obtained from the ant genome working group. The *Tribolium *genome and protein sequences were obtained from beetlebase. Protein sets and genomes for 12 *Drosophila *species were downloaded from flybase. Protein and genome data sets for *Anopheles*, *Aedes*, *Culex*, *Pediculus*, and *Iozides *were downloaded from vectorbase. *Bombyx *proteins and genome were downloaded from the silkworm genome database. The human genome was downloaded from UCSC, while human proteins were obtained from NCBI. The *C. elegans *genome and proteins were obtained from wormbase. Yeast proteins and genome were obtained from *Saccaromyces *Genome Database. *Nasonia *and *Acyrthosiphon *(pea aphid) genomes were obtained from HGSC, while their protein sets were obtained from NCBI. The latest version of the protein set and genome was used in all cases.

### Gene homology classification

We classified honey bee genes into 7 categories: orphans, Hymenoptera, social insect, Hymenoptera-conserved, insect specific, metazoan, and other. Orphans were genes for which we could find no homologs in any species, defining homology as a blast hit with an E value of less than 10^-4 ^[12-15]. Hymenoptera includes genes for which we could find at least one homolog in a Hymenopteran species, but no homologs anywhere else. Social insect means that the gene was found in all social insect genomes used in the study (*Linepethelia, Atta*, and *Pogonomyrmex*), but none other. Hymenoptera means the gene was found in all Hymenopteran species but none other. Insect specific means the gene was found in all insect genomes, but not in any non insect. Metazoan means the gene was found in all metazoans (with no reference to whether it is also present in single celled organisms). Thus, metazoan is the category that includes most conserved genes. Finally, 'other' included genes that did not show a pattern of homology that correlates with phylogeny or showed a taxonomic pattern that was not examined in this study.

Our procedure for classification was as follows. We used the current version of stand alone blast (2.23) with default parameters for all initial assignments. We began with the set of all protein translations from the honey bee OGS2. We used blastp to search for a homolog for each protein in each of the 27 other species-level protein sets. Those genes for which we could find no homolog in any alternative-spliced form became the gene set used for the next set of searches, which was to use tblastn to search for a homolog against the same species' genome. For each species we used all available genomic data. This ranged from one file for the entire genome for *Drosophila *and *Bombyx*, to separate files for scaffolds, contigs, and reads for the ants, and several other species. The results of this initial analysis (blasts against individual species proteins and genomes) were used to create 27 lists of each gene in the OGS2 that indicated whether it is present or absent in a particular species. This first stage of the process was conducted using in-house scripts. The 27 hashes were then uploaded into a database from which queries were made to determine the phylogenetic classification of each gene. All TRGs that emerged (orphans, Hymenoptera, social insect, Hymenoptera-conserved, and insect specific) were then searched against the current non redundant protein data base at NCBI using blastp. Several genes initially assigned to the various taxonomic categories were found in species such as *Bombus *(bumble bee), *Polistes *(paper wasp), daphnia, hydra, or chicken and moved to the correct classification.

### Gene characteristics

For each gene in 6 categories of conservation (orphan, Hymenoptera, Hymenoptera-conserved, social insect, insect-specific, and metazoan), we used scripts to determine protein length, gene length, GC content, number of exons, and whether the gene has transcript support. Gene length was parsed from gff files obtained from beebase, while transcript support was determined using stand alone tblastn, with word size = 20, seg turned off, ungapped setting, and E value cutoff of 10^-4^. Each gene was blasted against all honey bee ESTs (downloaded from NCBI). Changing the E value cutoff to 10^-8^, or lower, changes the number of alignments, but not the ratio between classes.

### Interproscan annotation

Functional annotations of genes in each taxonomically restricted category were determined using interproscan. For each gene all output from interproscan was collected and analyzed. Little data resulted from the searches of strongly taxonomically restricted genes, but much data was collected for insect specific genes.

### Gene duplication analyses

The goal of these analyses is to determine which TRGs have paralogs (in the honey bee) that are more conserved than the TRG. When this is true, it suggests that the TRG could have evolved by a gene duplication followed by rapid sequence divergence. Hence, we blasted all TRGs against all honey bee gene predictions and genes in OGS2. Those TRGs that had paralogs were then further analyzed to determine if any of the paralogs had homologs outside the original taxonomic class of the parent gene. For orphans, this meant at least one of the gene's paralogs had a homolog in another species. For genes in the classes Hymenoptera-conserved, Hymenopter-other, and Social Insect, one of a gene's paralogs had to have a homolog outside the Hymenoptera. For insect-specific proteins, this meant that a gene had a honey bee paralog that had a homolog outside the insects.

### Data sets from Grozinger et al [25], Alaux et al [27], and HPG genes

For each data set on genes involved in eusocial behavior or evolution we performed the same basic procedure. The goal was first to identify as many genes as possible from the ESTs used in the microarrays, and second to determine the taxonomic classification of each identified gene. We chose to focus on genes (proteins) only and not on ESTs for blast searches because EST alignments to species other than the one from which they were generated have a high error rate.

From Christina Grozinger we received a spreadsheet containing raw data from Grozinger et al [25]. This consisted of lists of ESTs/genes expressed at a higher level in queens relative to workers, and those expressed at a higher level in workers relative to queens (statistical methodologies given in Grozigner et al [25]). A third category of ESTs/genes non-differentially expressed was found by subtracting the first two lists from the complete list of ESTs/genes in the microarrary (also provided by Grozinger). From the supplemental information from Alaux et al [27] we obtained similar data for ESTs/genes expressed at a higher rate in nurses or foragers. Finally, from Gene Robinson we obtained a list of ESTs/genes expressed in the hypopharyngeal glands.

Each data set consisted of genes for which the original authors were able to align ESTs from the microarray to genes and unaligned ESTs. Our first step was to align as many of the unaligned ESTs to genes as possible. This is for two reasons. First, a new official gene set has been released since one of these studies, and second, the automated alignment procedure used in these studies would be unlikely to align ESTs to small genes (orphans are known to be small genes). This is because the automated alignment procedure used required that almost the entire EST align to a gene. As the ESTs averaged about 400 nucleotides and many orphans are genes shorter than 60 amino acids, this is impossible. ESTs can, however, be aligned to small genes manually. Hence, we first ran an automated search with a script and blastx to attempt to align each EST to either a member of the official gene set or an abinito prediction, using E value 10^-4 ^as the cutoff. We then manually aligned each positive hit, again using blastx, against the protein to ensure that the alignment was correct. Only ESTs with a large ungapped alignment to a single gene were considered matches. A list of all manual alignments is included in the supplemental information [Additional file 7: Supplemental Table S7]. In total, we manually aligned 51 additional ESTs that were expressed at higher rates in queens, 71 expressed higher in workers, 146 that were not differentially between queens and workers, 8 expressed higher in nurses, and 13 expressed higher in foragers.

## Authors' contributions

BRJ conducted the research and co-wrote the manuscript. NDT co-wrote the manuscript. All authors read and approved the final manuscript.

## Supplementary Material

Additional file 1 **Table S1 **List of insect specific proteins (excel file). Click here for file

Additional file 2 **Table S2 **Functional characteristics of insect specific proteins (excel file). Click here for file

Additional file 3 **Table S3 **Taxonomically restricted genes with paralogs (parent genes) that show wide conservation (excel file). Click here for file

Additional file 4 **Table S4 **Table showing the results from an analysis of the relationship between homology status and pattern of gene expression with different cut-off p-values for significance. Click here for file

Additional file 5 **Table S5 **Name and classification of genes expressed in the Hypopharyngeal gland (excel file). Click here for file

Additional file 6 **Table S6 **List of candidate taxonomically restricted genes involved in eusocial evolution (excel file). Click here for file

Additional file 7 **Table S7 **List of manual EST alignments to gene predictions (excel file). Click here for file

## References

[B1] Abouheif E Wray GA Evolution of the gene network underlying wing polyphenism in ants Science 2002 297 5579 249 252 10.1126/science.1071468 12114626

[B2] Toth AL Robinson GE Evo-devo and the evolution of social behavior Trends in Genetics 2007 23 7 334 341 10.1016/j.tig.2007.05.001 17509723

[B3] Smith CR Toth AL Suarez AV Robinson GE Genetic and genomic analyses of the division of labour in insect societies Nature Reviews Genetics 2008 9 10 735 748 10.1038/nrg2429 18802413

[B4] Robinson GE Genomics and integrative analyses of division of labor in honeybee colonies American Naturalist 2002 160 S160 S172 10.1086/342901 18707474

[B5] Whitfield CW Ben-Shahar Y Brillet C Leoncini I Crauser D LeConte Y Rodriguez-Zas S Robinson GE Genomic dissection of behavioral maturation in the honey bee Proceedings of the National Academy of Sciences of the United States of America 2006 103 44 16068 16075 10.1073/pnas.0606909103 17065327 PMC1622924

[B6] Johnson BR Division of labor in honeybees: form, function, and proximate mechanisms Behavioral Ecology and Sociobiology 2010 64 3 305 316 10.1007/s00265-009-0874-7 20119486 PMC2810364

[B7] West-Eberhard MJ Eder J and Rembold H The epigenetical origins of insect sociality Chemistry and Biology of Social Insects 1987 Munich: Verlag J. Peperny 369 372

[B8] West-Eberhard MJ Developmental Plasticity and Evolution 2002 Oxford: Oxford University Press

[B9] Linksvayer TA Wade MJ The evolutionary origin and elaboration of sociality in the aculeate hymenoptera: Maternal effects, sib-social effects, and heterochrony Quarterly Review of Biology 2005 80 3 317 336 10.1086/432266 16250466

[B10] Hunt BG Wyder S Elango N Werren JH Zdobnov EM Yi SV Goodisman MA Sociality is linked to rates of protein evolution in a highly social insect Molecular Biology and Evolution 2010 27 3 497 500 10.1093/molbev/msp225 20110264

[B11] Nelson CM Ihle KE Fondrk MK Page RE Amdam GV The gene vitellogenin has mutilple coordinating effects on social organization Plos Biology 2007 5 10.1371/journal.pbio.0050062 17341131 PMC1808115

[B12] Domazet-Loso T Tautz D An evolutionary analysis of orphan genes in Drosophila Genome Research 2003 13 10 2213 2219 10.1101/gr.1311003 14525923 PMC403679

[B13] Zhang G Wang H Shi J Wang X Zheng H Wong GKS Clark T Wang W Wang J Kang L Identification and characterization of insect-specific proteins by genome data analysis Bmc Genomics 2007 8 17407609 10.1186/1471-2164-8-93 PMC1852559

[B14] Toll-Riera M Bosch N Bellora N Castelo R Armengol L Estivill X Alba MM Origin of Primate Orphan Genes: A Comparative Genomics Approach Molecular Biology and Evolution 2009 26 3 603 612 10.1093/molbev/msn281 19064677

[B15] Mazza R Strozzi F Caprera A Ajmone-Marsan P Williams JL The other side of comparative genomics: genes with no orthologs between the cow and other mammalian species Bmc Genomics 2009 10 10.1186/1471-2164-10-604 20003425 PMC2808326

[B16] Khalturin K Hemmrich G Fraune S Augustin R Bosch TCG More than just orphans: are taxonomically-restricted genes important in evolution? Trends in Genetics 2009 25 9 404 413 10.1016/j.tig.2009.07.006 19716618

[B17] Lin HN Moghe G Ouyang S Iezzoni A Shiu SH Gu X Buell CR Comparative analyses reveal distinct sets of lineage-specific genes within Arabidopsis thaliana Bmc Evolutionary Biology 2010 10 10.1186/1471-2148-10-41 PMC2829037 20152032

[B18] Zdobnov EM von Mering C Letunic I Torrents D Suyama M Copley RR Christophides GK Thomasova D Holt RA Subramanian GM Comparative genome and proteome analysis of anopheles gambiae and Drosophila melanogaster Science 2002 298 5591 149 159 10.1126/science.1077061 12364792

[B19] Dai D Chen Y Chen S Mao Q Kennedy K Landback P Eyre-Walker A Du W Long M The evolution of courtship behaviors through the origination of a new gene in *Drosophila* Proceedings of the National Academy of Sciences of the United States of America 2008 105 21 7478 7483 10.1073/pnas.0800693105 18508971 PMC2396706

[B20] Khalturin K Anton-Erxleben F Sassmann S Wittlieb J Hemmrich G Bosch TCG A Novel Gene Family Controls Species-Specific Morphological Traits in Hydra Plos Biology 2008 6 11 2436 2449 10.1371/journal.pbio.0060278 PMC2586386 19018660

[B21] Fry BG Roelants K Winter K Hodgson WC Griesman L Kwok HF Scanlon D Karas J Shaw C Wong L Novel Venom Proteins Produced by Differential Domain-Expression Strategies in Beaded Lizards and Gila Monsters (genus Heloderma) Molecular Biology and Evolution 2010 27 2 395 407 10.1093/molbev/msp251 19837656

[B22] Winston ML The Biology of the Honey Bee 1987 Cambridge: Harvard University Press

[B23] Seeley TD The Wisdom of the Hive 1995 Cambridge: Harvard University Press

[B24] Johnson BR Linksvayer TA Deconstructing the superorganism: social physiology, groundplans, and sociogenomics Quarterly Review of Biology 2010 85 1 57 79 10.1086/650290 20337260

[B25] Grozinger CM Fan YL Hoover SER Winston ML Genome-wide analysis reveals differences in brain gene expression patterns associated with caste and reproductive status in honey bees (Apis mellifera) Molecular Ecology 2007 16 22 4837 4848 10.1111/j.1365-294X.2007.03545.x 17927707

[B26] Alaux C Le Conte Y Adams HA Rodriguez-Zas S Grozinger CM Sinha S Robinson GE Regulation of brain gene expression in honey bees by brood pheromone Genes Brain and Behavior 2009 8 3 309 319 10.1111/j.1601-183X.2009.00480.x 19220482

[B27] Quevillon E Silventoinen V Pillai S Harte N Mulder N Apweiler R Lopez R InterProScan: protein domains identifier Nucleic Acids Research 2005 33 116 120 10.1093/nar/gki442 PMC1160203 15980438

[B28] Weinstock GM Robinson GE Gibbs RA Worley KC Evans JD Maleszka R Robertson HM Weaver DB Beye M Bork P Insights into social insects from the genome of the honeybee Apis mellifera Nature 2006 443 7114 931 949 10.1038/nature05260 17073008 PMC2048586

[B29] Conant GC Wolfe KH Turning a hobby into a job: How duplicated genes find new functions Nature Reviews Genetics 2008 9 12 938 950 10.1038/nrg2482 19015656

[B30] Foret S Wanner KW Maleszka R Chemosensory proteins in the honey bee: Insights from the annotated genome, comparative analyses and expressional profiling Insect Biochemistry and Molecular Biology 2007 37 1 19 28 10.1016/j.ibmb.2006.09.009 17175443

[B31] Viljakainen L Evans JD Hasselmann M Rueppell O Tingek S Pamilo P Rapid Evolution of Immune Proteins in Social Insects Molecular Biology and Evolution 2009 26 8 1791 1801 10.1093/molbev/msp086 19387012

[B32] Lynch M Conery JS The evolutionary fate and consequences of duplicated genes Science 2000 290 5494 1151 1155 10.1126/science.290.5494.1151 11073452

[B33] Bilikova K Hanes J Nordhoff E Saenger W Klaudiny J Simuth J Apisimin, a new serine-valine-rich peptide from honeybee (Apis mellifera L.) royal jelly: purification and molecular characterization Febs Letters 2002 528 1-3 125 129 10.1016/S0014-5793(02)03272-6 12297291

[B34] Crailsheim K The flow of jelly within a honeybee colony Journal of Comparative Physiology B-Biochemical Systemic and Environmental Physiology 1992 162 8 681 689 10.1007/BF00301617

[B35] Drapeau MD Albert S Kucharski R Prusko C Maleszka R Evolution of the Yellow/Major Royal Jelly Protein family and the emergence of social behavior in honey bees Genome Research 2006 16 11 1385 1394 10.1101/gr.5012006 17065613 PMC1626640

[B36] Werren JH Richards S Desjardins CA Niehuis O Gadau J Colbourne JK Beukeboom LW Desplan C Elsik CG Grimmelikhuijzen CJP Functional and Evolutionary Insights from the Genomes of Three Parasitoid Nasonia Species Science 2010 327 5963 343 348 10.1126/science.1178028 20075255 PMC2849982

[B37] Sutherland TD Campbell PM Weisman S Trueman HE Sriskantha A Wanjura WJ Haritos VS A highly divergent gene cluster in honey bees encodes a novel silk family Genome Research 2006 16 11 1414 1421 10.1101/gr.5052606 17065612 PMC1626643

[B38] Katzav-Gozansky T Soroker V Ionescu A Robinson GE Hefetz A Task related chemical analysis of labial gland volatile secretion in worker honeybees (*Apis mellifera lingustica*) Journal of Chemical Ecology 2001 27 919 926 10.1023/A:1010330902388 11471944

[B39] Hölldobler B Wilson EO The Ants 1990 Cambridge: Harvard University Press

[B40] Oster GF Wilson EO Caste and Ecology in the Social Insects 1978 Princeton: Princeton University Press 740003

